# The mitochondrial genome of the Spanish honey bee, *Apis mellifera iberiensis* (Insecta: Hymenoptera: Apidae), from Portugal

**DOI:** 10.1080/23802359.2019.1693920

**Published:** 2019-12-09

**Authors:** Leigh Boardman, Amin Eimanifar, Rebecca Kimball, Edward Braun, Stefan Fuchs, Bernd Grünewald, James D. Ellis

**Affiliations:** a Honey Bee Research and Extension Laboratory, Entomology and Nematology Department, University of Florida, Gainesville, FL, USA;; b Department of Biology, University of Florida, Gainesville, FL, USA;; c Institut für Bienenkunde, Polytechnische Gesellschaft, Goethe-Universität Frankfurt am Main, Oberursel, Germany

**Keywords:** *Apis mellifera iberica*, A-lineage, NGS, mitogenome

## Abstract

The Spanish honey bee *Apis mellifera iberiensis*, had a mitochondrial genome of 16,560 bp. It consisted of 13 protein-coding genes, 22 tRNA genes, two rRNA genes, and an AT-rich control region. The sample was from Portugal and its mitogenome resembled those of the African (A)-lineage honey bee subspecies. It was most closely related to other North African honey bees, namely *A. m. sahariensis* and *A. m. intermissa*.

The Spanish honey bee, *Apis mellifera iberiensis* (Engel 1999), is found on the Iberian Peninsula (Spain and Portugal), as well as some nearby islands. Honey bees of this subspecies can have either African (A)-lineage or west Mediterranean and northwest European (M)-lineage mitochondrial haplotypes, with honey bees in the southwest of its range being A-lineage and those in the northeast being M-lineage (Smith et al. 1991; Cánovas et al. 2008; Chávez-Galarza et al. 2015). Here we sequenced an *A. m. iberiensis* worker bee sampled from Portugal (presumed to represent A-lineage *A. m. iberiensis*). The sample was obtained from the Ruttner Bee Collection at the Bee Research Institute in Oberursel (Voucher 1964, Dr. W.S. Sheppard, 1991, Evora, Portugal, 38°33 N 7°54 W). The GenBank accession number is MN585110.

Genomic DNA was extracted and quantified following Eimanifar et al. (2017) before genomic library preparation and next-generation sequencing (PE-150bp, Illumina Hi-Seq 3000/4000, San Diego, CA) were completed. The quality of sequencing reads was assessed using FastQC (Andrews 2010) and data were trimmed with Trimmomatic (Bolger et al. 2014). Stringent mapping was performed in Geneious Prime 2019.0.4 (Kearse et al. 2012) following Boardman et al. (2019) with the R1 paired trimmomatic output mapped to the reference genome with the highest pairwise identity – in this case *Apis mellifera sahariensis* (MF351881). The assembled mitogenome was annotated in mitos2 (Bernt et al. 2013) and manually adjusted in Geneious. Phylogenetic relationships were determined using a Mesquite v3.5 (Maddison and Maddison 2018) alignment of the 13 protein-coding genes (PCGs) and two ribosomal RNA (rRNA) regions and RAxML 8.2.10 GTRGAMMA model (1000 bootstrap replicates, -f a option, Stamatakis 2014) on CIPRES Science Gateway v. 3.3 (Miller et al. 2010). The *P*-distances were generated with PAUP 4.0a (Swofford 2003).

The mitogenome of *A. m. iberiensis* was 16,560 bp long and consisted of 43.2% A, 41.6% T, 9.6% C, and 5.6% G. The location of the 13 PCGs, two rRNAs, and 22 transfer RNAs (tRNAs) resembled arthropod mitogenomes, with nine PCGs (*nad2*, *co1*, *co2*, *atp8*, *atp6*, *co3*, *nad3*, *nad6*, and *cytb*) on the light strand, and four PCGs (*nad1*, *nad4*, *nad4l*, and *nad5*) and the two rRNAs on the heavy strand. The PCGs used four different start codons: ATT, ATG, ATA, or ATC, and all 13 ended with the TAA stop codon. *Atp8* and *atp6* overlapped, with 19 shared nucleotides. The 16S rRNA was 1,324 bp with 84% AT, while 12S rRNA was 785 bp with 81.1% AT. The 22 tRNAs were 63 to 78 bp long with the shortest tRNA being glutamate and serine, and the longest, threonine.

Phylogenetically, this sample was confirmed as being part of the A-lineage (Figure 1). It was most closely related to North African subspecies *A. m. sahariensis* (*P*-distance: 0.00183) and *Apis mellifera intermissa* (*P*-distance: 0.00198). Southern African subspecies, *Apis mellifera scutellata*, *Apis mellifera adansonii*, *Apis mellifera capensis*, and *Apis mellifera monticola*, have *P*-distances greater than 0.004. The *A. mellifera* subspecies with the highest *P*-distance from *A. m. iberiensis* was *Apis mellifera mellifera* (*P*-distance: 0.01395).

**Figure 1.**
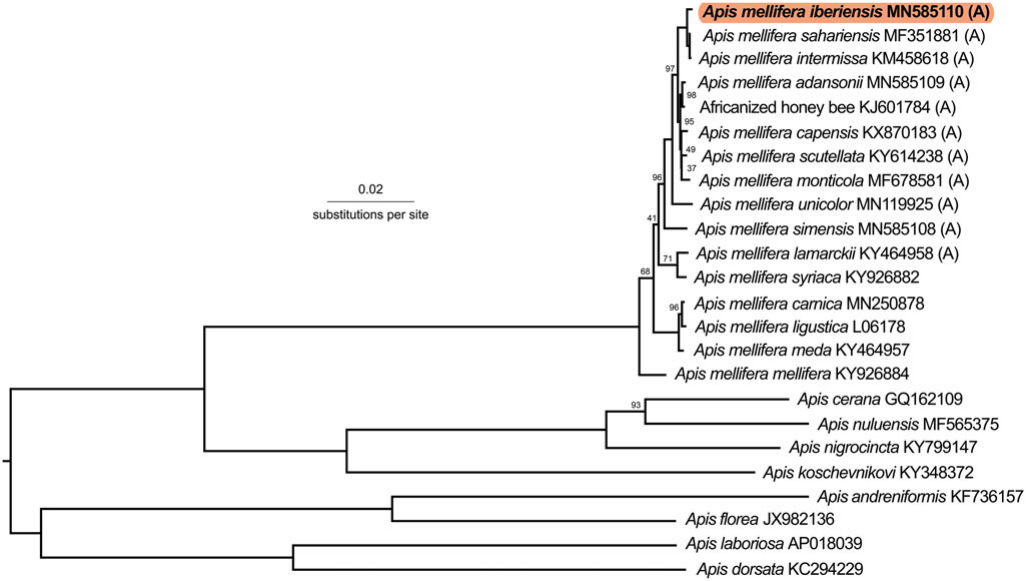
Phylogenetic tree showing the relationship between *Apis mellifera iberiensis* (GenBank: MN585110) and 23 other honey bee mitochondrial genomes. The sequences from 13 protein-coding genes and two rRNAs were used to build the tree. The tree is midpoint rooted. Node labels indicate bootstrap values and unlabeled lineages are 100%. The GenBank accession numbers are given after the species names. Subspecies of African (A)-lineage are denoted with (A).
